# Deficit in Adipose Differentiation in Mesenchymal Stem Cells Derived from Chronic Rhinosinusitis Nasal Polyps Compared to Nasal Mucosal Tissue

**DOI:** 10.3390/ijms21239214

**Published:** 2020-12-03

**Authors:** Emanuela Chiarella, Nicola Lombardo, Nadia Lobello, Giovanna Lucia Piazzetta, Helen Linda Morrone, Maria Mesuraca, Heather Mandy Bond

**Affiliations:** 1 Laboratory of Molecular Haematopoiesis and Stem Cell Biology, Department of Experimental and Clinical Medicine, University “Magna Græcia”, 88100 Catanzaro, Italy; emanuelachiarella@unicz.it (E.C.); helen.morrone@gmail.com (H.L.M.); 2 Otolaryngology Head and Neck Surgery, Department Medical and Surgical Sciences, University “Magna Græcia”, 88100 Catanzaro, Italy; nlombardo@unicz.it (N.L.); nadialobello@gmail.com (N.L.); giovannapiazzetta82@gmail.com (G.L.P.)

**Keywords:** nasal polyps, mesenchymal cells, adipocyte differentiation, apoptosis

## Abstract

Chronic rhinosinusitis of the nasal mucosa is an inflammatory disease of paranasal sinuses, which causes rhinorrhea, nasal congestion, and hyposmia, and in some cases, it can result in the development of nasal polyposis. Nasal polyps are benign lobular-shaped growths that project in the nasal cavities; they originate from inflammation in the paranasal mucous membrane and are associated with a high expression of interleukins (IL)-4, IL-5, IL-13, and IgE. Polyps derive from the epithelial–mesenchymal transition of the nasal epithelium resulting in a nasal tissue remodeling. Nasal polyps from three patients with chronic rhinosinusitis as well as control non-polyp nasal mucosa were used to isolate and cultivate mesenchymal stem cells characterized as CD73^+^, CD90^+^, CD105^+^/CD14^−^, CD34^−^, and CD45^−^. Mesenchymal stem cells (MSCs) cultures were induced to differentiate toward adipocytes, where lipid droplets and adipocyte genes PPARγ2, ADIPO-Q, and FABP4 were observed in control non-polyp nasal mucosa-derived mesenchymal cells but were scarcely present in the cultures derived from the nasal polyps, where apoptosis was evident. The modulation of the response to adipogenic stimulus in polyps represents a change in the molecular response that controls the cascade required for differentiation as well as possible means to specifically target these cells, sparing the normal mucosa of the nasal sinuses.

## 1. Introduction

Chronic rhinosinusitis (CRS) is a frequent medical condition that adversely affects the quality of life of patients causing nasal obstruction and congestion, nasal drainage, facial pain or pressure, and hyposmia/anosmia [1], and it is supported by heterogeneous inflammatory processes. CRS can be divided into cases without nasal polyposis (CRSsNP) and CRS with nasal polyposis (CRSwNP) [2]. The cases can be categorized into different subtypes depending on the Th2 versus Th1/Th17 cell response together with the sets of cytokines, chemokines, and growth factors involved [3] and resulting in a balance of endotype and phenotypes, as described in the guidelines of the EPOS 2020 [4,5]. Nasal polyps are noncancerous lesions originating from the mucosa of the nasal sinuses. They hang down in a manner similar to teardrops and appear as soft and painless growths. The development of nasal polyps is the consequence of chronic inflammation affecting the mucous membranes in the paranasal sinuses and is caused by a multitude of stimuli including viruses, bacteria, fungi, and allergens [6]. Additionally, it can be associated with several risk factors: allergies, drug sensitivity, asthma, exposition to airborne irritants, immune system disorders, viral infections, or deviated septum or enlarged turbinates. When the growths become large, they can cause breathing difficulties, a reduced sense of smell or taste, and recurrent infections [1].

CRSwNP and CRSsNP conditions have different immunological cytokine profiles that are also associated with geographical and race variations [7]. The inflammatory pattern of CRSwNP has revealed a tissue eosinophilia mediated by Th2 cytokines and is associated to a high asthma prevalence rate. CRSwNP, compared to CRSsNP, shows increased levels of Th2 mediators including interleukin(IL)-4, IL-5, IL-13, IgE and eotaxin-2 [4,8]. These cytokines can be therapeutic targets using blocking monoclonal antibodies [9,10,11,12,13].

The tissue remodeling in nasal polyps is traceable to epithelial–mesenchymal transition (EMT) a biological process in which epithelial cells, undergoing biochemical changes, acquire mesenchymal and fibroblast-like properties with reduced intercellular adhesion and increasing migratory capacity, invasiveness as well as elevated resistance to apoptosis [14]. These nasal polyps can be a novel source of mesenchymal stem and progenitor cells, which can be isolated from small surgical biopsies [15].

Mesenchymal stem cells derived from polyps (NPO-MSCs) meet the basic criteria for typical MSCs, including (I) adherence to plastic culture plates; (II) high expression for the cell surface markers CD90, CD73, and CD105 and the absence of MHC-II (class of major histocompatibility complex), CD45, CD34, CD14, or CD11b surface antigens; and (III) multipotential ability to differentiate toward mature osteoblasts, adipocytes, and chondroblasts in vitro [16,17,18,19,20].

Here, we have isolated and characterized mesenchymal stem cells from nasal polyps (NPO-MSCs) by polypectomy endoscopic surgery procedures and from non-polyp nasal mucosa tissue of the same patient (CTL-MSCs). These nasal MSCs have similar phenotypes and growth rates and express CD90, CD73, and CD105. Results show that under adipogenic stimuli, NPO-MSCs compared to nasal tissue-derived MSCs (CTL-MSCs) are hardly able to differentiate into adipocytes and instead undergo apoptosis.

Our data provide new biological and molecular information on the behavior of NPO-MSCs during differentiation, which is useful for investigating molecular mechanisms underlying the development of nasal polyps, including mesenchymal–epithelial transition. The response to adipocyte differentiation stimulus where the NPO-MSCs fail to form adipocytes and cell death is activated by apoptosis represents a targeting mechanism for the polyps not affecting the non-polyp nasal mucosa tissue of the same patient.

## 2. Results

**Figure 1:**
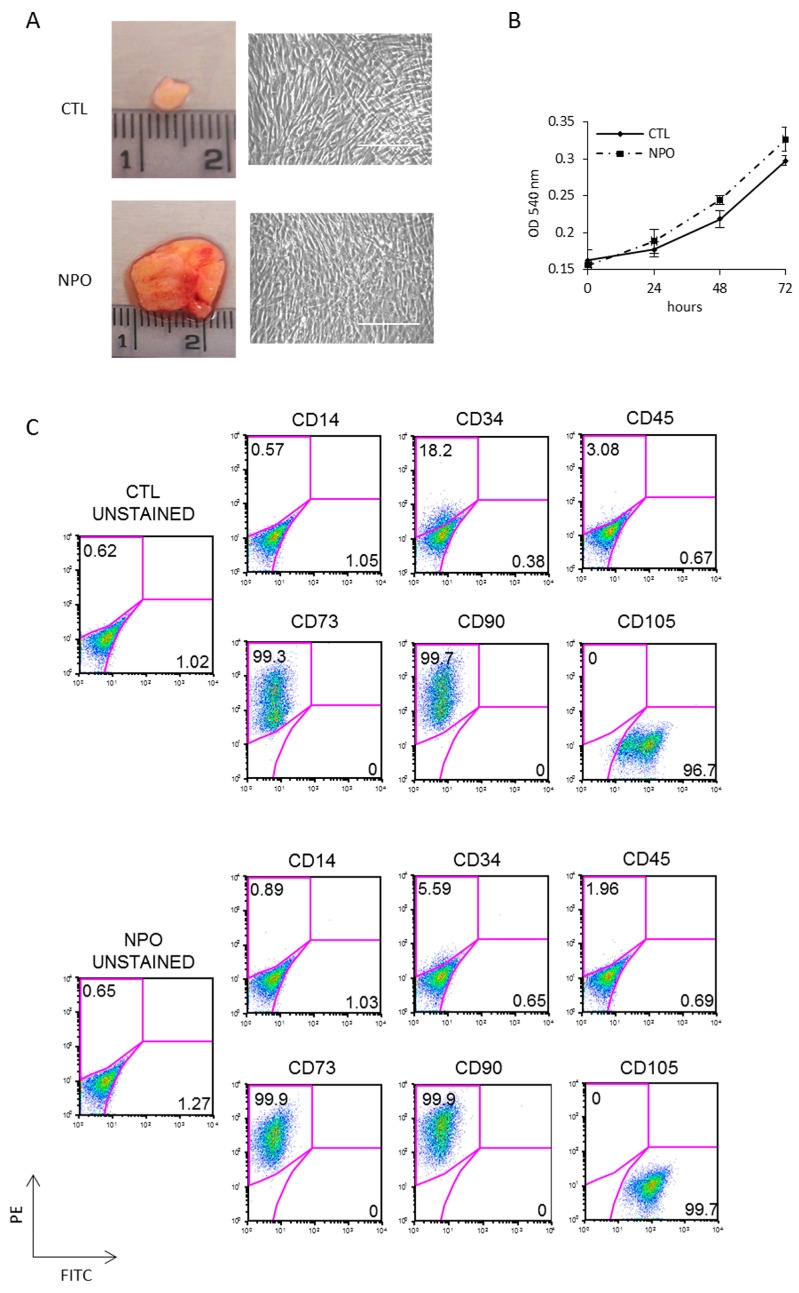
Isolation and characterization of mesenchymal stem cells from nasal polyps and nasal mucosa tissue. (**A**) Representative nasal biopsies from a patient with chronic rhinosinusitis (CRS). Polyps-derived mesenchymal stem cells were obtained by mechanical dissociation and collagenase digestion of the tissues. The morphology of adherent cells after three passages was observed by phase contrast microscope (20×) Scale bar indicates 100µm. (**B**) The growth rates of mesenchymal stem cells from non-polyp nasal mucosa tissue (CTL-MSCs) and mesenchymal stem cells from nasal polyps from the same patient (NPO-MSCs) were analyzed by 3-(4,5-dimethyl-2-thia-zolyl)-2, 5-diphenyl-2H-tetrazolium bromide (MTT) assays. (**C**) The phenotypic pattern for CD73, CD90, and CD105 expression was investigated by FACS analysis.

Polypectomy endoscopic surgery was performed on three different CRSwNP patients; to remove polyps, this treatment will give temporary relief, but the prediction is that they will reoccur within months, as the primary cause has not been addressed. Mesenchymal stem cells were purified from nasal polyps (NPO-MSCs), which are typically translucent pink with a lobus grape-like structure; an example is shown in Figure 1A together with a small piece of nasal mucosa tissue used as a control. CTL-MSCs and NPO-MSCs were derived from surgical biopsies of three different patients with CRSwNP, and clean monolayers of cells were obtained after 3 weeks of culture (Figure 1A). These cells were tested for cellular proliferation and were found to have similar growth rates being derived either from the control or polyp tissues in all three of the patients analyzed (Figure 1B). Cultures were characterized for mesenchymal phenotype by FACS being uniformly positive for the mesenchymal markers CD73, CD90, and CD105 and negative for CD14, CD34, and CD45 (Figure 1C).

**Figure 2:**
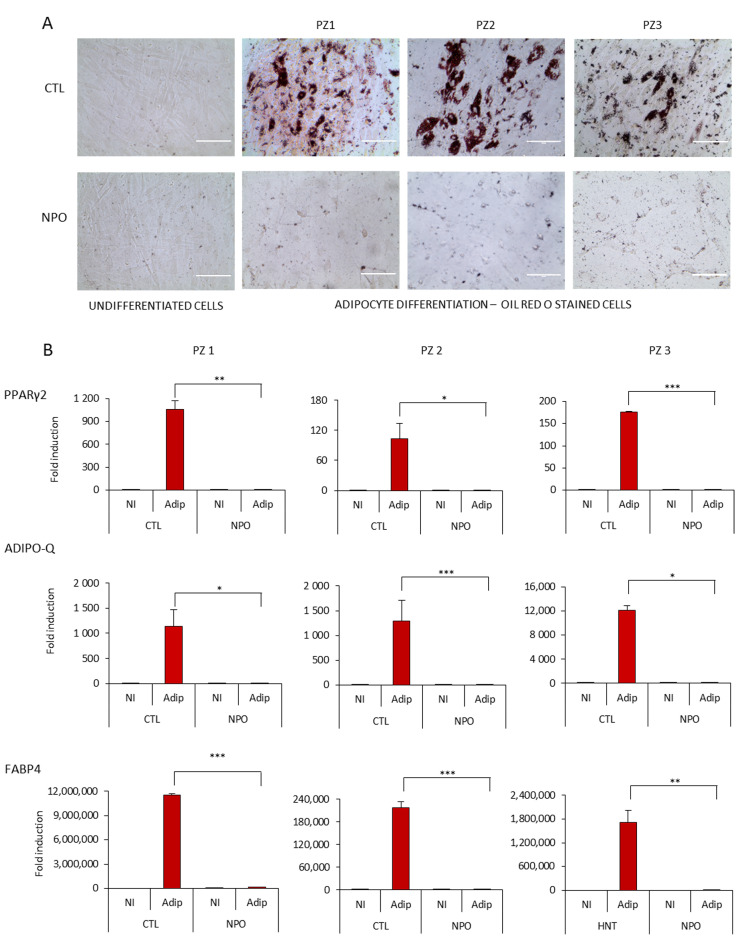
Adipocyte differentiation of CTL-MSCs and NPO-MSCs. (**A**) Mesenchymal stem cells were cultivated in either proliferation MesenPRO RS™ medium (non-induced, NI) or in STEM PRO^®^ adipogenesis differentiation medium (Adip) for 14 days, stained with Oil Red O, and observed by phase contrast microscopy (20×) Scale bar indicates 1000µm. The assay was performed in triplicate. (**B**) PPARγ2, ADIPO-Q, and FABP4 expression were quantified by RT-Q-PCR after 10 days of adipocyte differentiation. The gene expression was normalized for the housekeeping gene GAPDH (Glyceraldehyde-3-phosphate dehydrogenase). Data are represented as means ± SD from three different experiments performed in duplicate (* *p* < 0.05; ** *p* <0.001; *** *p* <0.0001).

Considering the capacity of mesenchymal cells to differentiate particularly into adipocytes, we tested both the nasal mucosa tissue-derived mesenchymal stem cells CTL-MSCs and the NPO-MSCs with standardized adipogenesis differentiation medium. It was found that after 14 days, the control nasal tissue-derived MSCs had a significant number of typical adipocytes where by phase contrast, lipid droplets were visible in many cells that were positive for Oil Red O staining (Figure 2A). However, it was notable that the NPO-MSCs scarcely formed lipid droplets with only a minimal amount of Oil Red O staining, and their morphology had changed with a spinodal-like appearance with less cell contacts. 

Adipogenesis requires the sequential activation of key transcription factors resulting either in an induction of genes involved in lipid metabolism or an inhibition by transcription factors that can block the onset of differentiation [21]. These nasal MSCs cultures were tested for the modulation of the master transcription factor for adipogenesis PPARγ2 (peroxisome proliferator-activated receptors) [22] (Figure 2B), which was upregulated in the CTL-MSCs during differentiation, but not from polyps. Similarly, markers of fat metabolism—fatty acid binding protein 4 (FABP4), a carrier protein for fatty acids [23], and adiponectin (Adipo-Q) [21], which is exclusively expressed in adipose tissue being present in the plasma acting as an adipokine controlling fat metabolism and insulin sensitivity—were also induced from non-existent to high levels in the control nasal MSCs during differentiation, but they were only induced to a minimal level in the NPO-MSCs.

**Figure 3:**
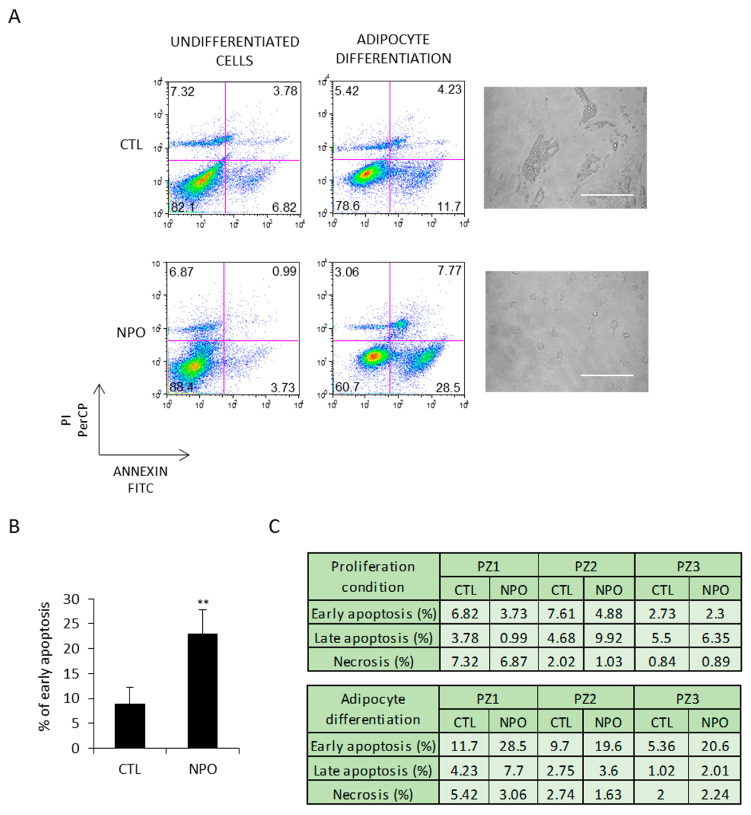
NPO-MSCs undergo apoptosis when cultured in adipocyte differentiation medium. (**A**) CTL-MSCs and NPO-MSCs were cultivated in either proliferation conditions (non-induced CTL) or in adipocyte differentiation medium. Images are in phase contrast and scale bar indicates 100 µm. Cells were stained for both annexin V and PI to evaluate early apoptosis, late apoptosis, and necrosis and analyzed by FACS. The results of early apoptosis are represented as means ± SD from three different experiments and (**B**,**C**) the percentage of early apoptosis and late apoptosis/necrosis are indicated in the table for each patient (** p < 0.001).

These cells were additionally tested in parallel for apoptosis. After 5 days, early apoptosis, analyzed by annexin V and PI staining, was evident in the cells derived from the nasal polyps and minimally in those from the CTL-MSC (Figure 3A). This was the case for all the three patients with control, and polyp-derived nasal MSCs and was at least almost three times more than evident in that from the nasal polyp-derived cells (Figure 3B). Late apoptosis and necrosis were only present at practically background levels, as reported in the table shown in Figure 3C.

## 3. Discussion

Mesenchymal stem cells purified from adipose tissue (ADSCs) and bone marrow (BM-MSCs) are known for their potentiality to differentiate in multi-lineages toward mature osteoblasts, adipocytes, chondroblasts, and neuronal-like cells [24,25,26]. Diverse experimental cellular tissues have been explored to obtain mesenchymal cells with potential for inherent differentiation, which may result into unique phenotypes. The use of adult somatic cells that can transdifferentiate can avoid using embryonic stem cell systems, which raises ethical issues. Nasal polyps are relatively frequent in CRS and have to be surgically removed periodically. They represent a discarded tissue for potential experimental and clinical exploration. 

In this manuscript, mesenchymal cells from nasal polyps from three different CRS patients (NPO-MSCs) are compared to MSCs derived from nasal mucosal tissue (CTL-MSCs) for their ability to differentiate toward adipose tissue. Even though the NPO-MSCs were characterized as having the mesenchymal markers CD73, CD90, and CD105 similar to CTL-MSCs, we showed that they have a considerably reduced ability to form characteristic lipid-filled adipocytes and undergo a significant degree of early apoptosis. Instead, the CTL-MSCs form typical adipocyte cultures with groups of cells staining strongly for Oil Red O. This is similar to those found stimulated with adipocyte differentiation medium derived from ADSCs cells [27,28,29].

Previous studies by Cho KS et al. [30] have used normal nasal tissue from healthy patients as a source of mesenchymal progenitor cells (MPCs) equivalent to MSCs from paranasal sinus mucosa and compared them with properties of MPCs of other tissue sources within the sinonasal cavity. MPCs were in the maxillary sinus (MS) and ethmoid sinus (ES) as well as the inferior turbinate (IT) and tonsils from the normal nasal cavity. These cultures from MS-, ES-, IT-, and T-MPCs yielded mesenchymal cells with similar morphologies and phenotypes, which are capable of distinct adipogenic, osteogenic, and chondrogenic differentiation abilities. The colony-forming potential and proliferation capability of ES-MPCs were distinctly higher than other MPCs.

Nasal polyp MSCs-derived cells, described by Cho JS et al. [17], can be induced to differentiate toward osteogenic, adipogenic, chondrogenic, and neural-like cells. Chondrocytic and osteogenic cells have robust colorations (alcian blue and alizarin red), whereas the adipocyte Oil Red O staining was evident, but the cells lacks translucent lipid droplets that can be found from ADSC or BM-MSCs. There were increases in FABP4 and PPARγ2 mRNAs [17] in induced cells, but the levels of expression are difficult to compare with other sources of MSCs, as the increases are as fold above a negative background. Additionally, neural induction was observed after 3 weeks by antibody staining of Class III B tubulin and the neural filaments NF-L and NF-H. In this case [17], the differentiation was compared to the non-induced proliferating polyp cells rather than with normal nasal tissues.

Gene expression tools and proteomic analysis are substantially contributing to clarify the features of mesenchymal stem cells and the protein cross-talk responsible for stemness [16,24,31]. MSCs were isolated [16] from nasal polyp tissue, and their gene expression profile and immune phenotype were compared with BM-MSCs. These MSCs cells were both found to have characteristic features of MSCs, expressing the cell surface antigens CD73, CD90, CD105, and ability to differentiate toward osteocytes and adipocytes [24]. Nasal PO-MSCs expressed higher levels of stem cells specific markers (CD133 and ABCB1), while BM-MSCs showed an elevated expression of cytokines and growth factors (FGF10, KDR, and GDF6), and PO-MSC displayed genes related to matrix remodeling process as found in polyps. Several MSCs immune-associated membrane markers CD117, HLA-DR, PD-L1, and PD-L2 were not expressed in PO-MSCs but were highly present in BM-MSCs. These markers are mainly associated with the immunoregulatory capacity of MSCs. 

These observations indicate that a classification as mesenchymal stem cells only partly defines the cell type, which will retain features and genes derived from the original tissue [24,26]. Adipocyte differentiation, although evident, has several limitations in terms of quantification: although it can be clearly seen by phase contrast and stained by Oil Red O, the number of adipocytes forming is difficult to estimate, as the adipocytes develop in patches rather than covering the entire dish/well. It is also difficult to compare absolute PCR gene expression data for adipocyte gene expression as the induction is from nothing to very high levels. This aspect is further complicated by different controls being used in the published manuscripts discussed, nasal polyp MSCs vs. MS-, ES, IT-, and T-MPCs mesenchymal cells [17,30], nasal polys vs. BM-MSCs [16], and here, nasal polyps (NPO-MSCs) vs. nasal mucosal tissue (CTL-MSCs) from the same patient. Additionally, the interpretation of these results is limited, considering that the majority of protocols use a commercial mix of differentiation stimulants from Gibco, Millipore, and here StemPro^®^ adipogenesis medium from Thermo Fisher Scientific (Monza, Italy), which on the one hand standardizes the procedure and gives a high probability of the differentiation working; however, it precludes the determination of which biomolecule/concentration/solvent is critical in the cocktail in a particular cellular setting.

## 4. Materials and Methods

### 4.1. Mesenchymal Stem Cells (MSCs) Isolation and Culture

MSCs cells were obtained from nasal polyps (0.7–0.9 cm) of CRS patients (CRSwNP) and controls from the same patient of nasal mucosal tissue (0.2–0.3 cm), which was non-polypoid. Cells were extracted by mechanical dissociation, using a scalpel blade followed by enzymatic digestion with Collagenase IV 1 mg/mL for 4–12 h at 37 °C. Cells were washed in PBS (Phosphate-buffered saline) and filtered through 70 µm filters and adherent cells were cultivated in tissue culture treated 6 wells plates in MesenPRO RS^TM^ medium (cat no. 12746-012 Thermo Fisher Scientific), 1× Glutamax (Life Sciences, Monza, Italy), 50U of penicillin/50 μg streptomycin/mL and gentamicin 20 μg/mL and then incubated at 37 °C in 5% CO_2_. After three passages, a uniform monolayer of adherent cells was obtained. Informed consent was obtained from donors given by patients.

### 4.2. Flow Cytometry and Apoptosis

MSCs cells were stained with fluorescent-conjugated antibodies for CD14, CD34, CD45, CD73, CD90, and CD105 (Miltenyi Biotec S.r.l., Bologna) by incubating with each antibody for 30 min, in the dark, at 4 °C and washed with PBS. Cells were re-suspended in 300 μL of PBS, acquired on the BD FACscan™ II, and data were analyzed with FlowJo software 8.8.6 (Becton Dickinson, Milan, Italy) [32].

### 4.3. Annexin V/Propidium Iodide (PI) Staining Assay

Apoptosis was evaluated using the kit from Life Technologies (Monza, Italy), an FITC (Fluorescein isothiocyanate) Annexin V apoptosis detection kit with Propidium Iodide (PI) staining after 5 days of culture, according to the manufactures instructions. Briefly, after 5 days of culture, 5 × 10^5^ cells were washed once with cold PBS and resuspended in 100 µL of binding buffer containing 5 µL of annexin V–FITC and 1 µL PI (100 µg/mL) for 15 min in the dark at room temperature. The samples were re-suspended in 400 µL of annexin V binding-buffer diluited to 1× with H_2_O for Flow Cytometry analysis (BD FACScan II™). The percentage of cells in early apoptosis or late apoptosis/necrosis was evaluated by gating annexin V^+^/PI^−^ and annexin V^+^/PI^+^ labeled cells, respectively, using FlowJo software 8.8.6 [33].

### 4.4. Cell Proliferation Assay

Cell proliferation was measured by the MTT assay which uses tetrazolium salt (3-(4,5-dimethyl-2-thia-zolyl)-2, 5-diphenyl-2H-tetrazolium bromide). Briefly, 5 × 10^2^ cells/well were seeded in 100 µL of MesenPro RS™ onto 96-well plates and assayed one to three days after incubation. The cells were treated with 10 μL of MTT reagent (5 mg/mL) (Sigma Aldrich, Milan, Italy) at 37 °C for 90 min and then treated with the addition of 110 µL of isopropanol prepared with 0.08 N HCl, which was added to block the reaction by cell lysis and solubilization of the formazan crystals. The colorimetric reaction was detected at 590 nm using the spectrofotometer Glomax (Promega, Milan, Italy). The absorbance registered was directly proportional to the number of viable cells [34].

### 4.5. Adipocyte Differentiation

MSCs after the third passage cells were seeded in 12-well plates at the density of 1 × 10^4^ cells/cm^2^. To induce differentiation, confluent cells were incubated for 14 days with STEM PRO^®^ adipogenesis differentiation medium (Thermo Fisher Scientific Cat No A1007001, previously marketed by Invitrogen, Lifetecnologies and Gibco, Monza, Italy). Control cells were cultured for proliferation in MesenPRO RS™ medium (Thermo Fisher Scientific) [29] representing non-induced (NI) MSCs controls.

### 4.6. Oil Red O Staining

Cells were washed twice with PBS and fixed with precooled 10% formaldehyde for 15 min at −20 °C. After fixation, the cells were washed twice with PBS, stained for 30 min at room temperature in freshly diluted Oil Red O Solution 0.5% in isopropanol diluted at 3:2 with distilled water, filtered with a 0.45 µm filter (O1391-Sigma), and then washed twice with distilled water. Oil Red O is a lysochrome (fat-soluble dye) diazo dye used for the selective staining and detection of neutral triglycerides and lipids contained in lipid droplets of cultured cells. Images were acquired using a bright-field microscope (EVOS M5000 Cell Imaging System, Life Technologies) [29].

### 4.7. RNA Isolation, Reverse Transcription and Quantitative RT-PCR

The TRI Reagent was used for RNA extraction (Sigma-Aldrich) and quantified with the NanoDrop 2000/2000c Spectrophotometer (Thermo Fisher Scientific) and the quality was monitored using 1.5% agarose gels run in MOPS buffer, pH 7.1 (0.4 M MOPS (3-(*N* morpholino)propanesulfonic acid), 0.1 M NaAc, 20 mM EDTA (Ethylenediaminetetraacetic acid)), and 10% formaldehyde. cDNA was synthesized from 1 µg RNA using SuperScript III reverse transcriptase at 42 °C and 2.5 µM random hexamers (Life Technologies). Quantitative RT-PCR (Q-RT-PCR) was performed using the SYBR™ Green master mix (Bio-Rad, Milan, Italy) and the iQ5 multicolor detection system (Bio-Rad). One cycle of 3 min at 95 °C was followed by 45 cycles of 10 s at 95 °C, 10 s at 60 °C, and 20 s at 72 °C, followed by a melting curve. mRNA levels are normalized to GAPDH expression as described in [35], and 2^−ΔΔCt^ values calculated. Relative gene expression was determined using the comparative threshold cycle Ct method, normalizing for housekeeping genes (*GAPDH*) such that the average the expression ratio was calculated as 2^−ddCt^. The primers for adipocyte specific gene amplification were: PPARγ2, FWD CCTATTGACCCAGAAAGCGATT, REV CATTACGGAGAGATCCACGGA); FABP4 (FWD TGGGCCAGGAATTTGACGAA, (REV GACGCATTCCACCACCAGTT); ADIPO-Q, FWD AGGGTGAGAAAGGAGATCC, REV GGCATGTTGGGGATAGTAA.

### 4.8. Statistics

All results are reported as mean ± standard deviation (SD). Statistical analysis was performed using *t*-test (* *p* < 0.05 were considered statistically significant).

## 5. Conclusions

Here, we compared the ability of NPO-MSCs and CTL-MSCs from patients to differentiate into mature adipocytes. Although NPO-MSCs and CTL-MSCs show the same the typical antigen expression pattern of mesenchymal stromal cells, when they are cultivated under adipocyte conditions, they show different fates. CTL-MSCs were able to differentiate into mature adipocytes characterized by roughly spherical shape and ectopic fat deposits. Instead, the NPO-MSCs were apparently committed only to preadipocytes and underwent growth arrest and consequent apoptosis.

In this study, we highlight that an adipocyte differentiation medium induces apoptosis in NPO-MSCs sparing control nasal mucosal tissue. The underlying mechanisms remain to be investigated and need further study to clarify the pathways involved which induce and sustain the programmed cell death programmed process.

## Abbreviations

CRS	Chronic rhinosinusitis
CRSsNP	CRS without nasal polyposis
CRSwNP	CRS with nasal polyposis
MHC-II	class of major histocompatibility complex
FACS	Fluorescence-activated Cell Sorting
PPARg2	peroxisome proliferator-activated receptors
FABP4	fatty acid binding protein 4
ADIPO-Q	adiponectin
ADSCs	Adipose-derived stem cells
MPC	Mesenchymal progenitor cells
ABC1	ATP Binding Cassette Subfamily A Member 1
FGF10	Fibroblast Growth Factor 10
KDR	Kinase Insert Domain Receptor
GDF6	Growth Differentiation Factor 6
HLA-DR	human leukocyte antigen-D related
PDL1-2	Programmed death-ligand 1-2
EDTA	Ethylenediaminetetraacetic acid
FITC	Fluorescein isothiocyanate
GADPH	Glyceraldehyde-3-phosphate dehydrogenase
MOPS	3-(N-morpholino)propanesulfonic acid
PBS	Phosphate-buffered saline
MesenPRO RS™	Mesenchymal Proliferation reduced serum Trade Mark
